# Plant cell–inspired colon-targeted cargo delivery systems with dual-triggered release mechanisms

**DOI:** 10.1126/sciadv.adt2653

**Published:** 2025-05-14

**Authors:** Anran Mao, Anna C. Gebhard, Nazanin Z. Ezazi, Aseem Salhotra, Anastasia V. Riazanova, Ravi Shanker, Lars Wågberg, Line Hagner Nielsen, Anna J. Svagan

**Affiliations:** ^1^ Department of Fibre and Polymer Technology, KTH Royal Institute of Technology, Teknikringen 56, 100 44 Stockholm, Sweden.; ^2^ Department of Health Technology, The Danish National Research Foundation and Villum Foundation's Center IDUN, Technical University of Denmark, 2800 Kgs. Lyngby, Denmark.

## Abstract

Plant cells represent smart cargo carriers with great socioeconomic potential in oral drug delivery applications. The two exterior barriers, featuring a rigid cell wall and a dense plasma membrane, are unique with complementary structural, mechanical, and chemical properties. Current strategies for producing therapeutic drugs within plant cells for oral delivery are efficient, but largely limited to recombinant pharmaceutical proteins, and involve complex genetic modification of plants. To address this, we engineer plant cell–inspired delivery systems with cellulose nanofiber–based shells and lipid layers through a bottom-up assembly strategy, which offers greater flexibility to encapsulate nonprotein compounds and nanoparticles. Notably, the layered shell structure resists degradation in acidic environments, and two barriers respond differently to external stimuli in simulated gastrointestinal medium, resulting in size-dependent dual-triggered release mechanisms. The cytocompatibility was shown by incubation with Caco-2 cells. Our results open avenues for developing next generation of bioinspired oral delivery systems for multisite-specific gastrointestinal release in a low-cost and sustainable manner.

## Teaser

Synthetic plant cells with cell wall and plasma membrane mimics are developed for multisite gastrointestinal cargo release.

## INTRODUCTION

Plant cells are essentially “capsule units” with a complex layered shell structure (*1*, *2*). The shell constitutes of an organized cell wall and a lipid-based plasma membrane, which combined form the first line of defense by protecting and regulating the passage of different types of molecules, thereby enabling various specific internal cellular functions. The primary cell wall, composed of cellulose microfibrils and fibril aggregates embedded in a polysaccharide matrix of pectin and hemicellulose, provides a rigid skeleton that offers mechanical stability, and accommodates the high turgor pressure in plant cells (*3*–*5*). This wall distinguishes plant cells from animal cells, being porous and highly permeable to small molecules (*6*). In contrast, the plasma membrane acts as a selective barrier, controlling and regulating the passage of substances in and out of the plant cell via active protein transporter pumps (*7*, *8*).

Over the years, plant cells have been evaluated as potential candidates for drug delivery, in particular for oral drug delivery (*9*, *10*). Apart from the good biocompatibility, the plant cell wall protects the encapsulated drug from the harsh environment of the stomach (low pH and enzymes) as the polymers of the (primary) cell wall—cellulose, pectin, and hemicellulose—are not affected (*11*). Only the gut microbes in the colon produce enzymes (e.g., glucosidase and pectinase), which can break down the wall in plant tissue cells (vegetables and fruit), leading to a release of internal content (*12*, *13*). This facilitates targeted drug delivery to the colon. Recent advances in plant molecular farming, using genetically engineered plants to express recombinant pharmaceutical proteins that are naturally bioencapsulated in plant cells, have been demonstrated for oral delivery application. However, cellular engineering of plant cells is limited to protein-based drugs, leaving great challenges in delivering nonprotein-based compounds within natural plant cells (*14*–*16*). Thus, mimicking specifically the structural and functional features in natural plant cells through synthetic pathways offers a greater flexibility for encapsulation and presents a promising strategy to address these challenges.

Current research primarily focuses on synthetic animal cells (lipid-based carriers for targeted delivery) with much less effort directed toward plant-based approaches (*17*–*20*). Colonic delivery can be effective for drug absorption of for example peptide drugs, and local drug delivery to the colon is of high importance in diseases such as colon cancer and inflammatory bowel diseases (*21*). However, orally administered lipid-based drug carriers have drawbacks when colonic drug delivery is the target because the lipid digestion occurs prior to reaching the colon, with most absorption taking place in the small intestine (*22*). Recent studies have reported the strategy of coating the liposomes with polymers such as chitosan and PVA (polyvinyl alcohol) for enhanced protection (*23*–*25*). Apart from this, there are several pH-sensitive drug delivery systems that dissolve between pH 6 to 7 (*26*, *27*). However, the pH in the different parts of the GI tract fluctuates between individuals due to natural variations, which might cause issues such as premature or delayed drug release. Enzymatically triggered release mechanisms, which rely on colonic bacteria, represent an alternative approach, offering colon-targeted drug delivery. Still, these systems are generally limited to the colon and face challenges in achieving multisite release (*28*, *29*). In this study, we investigate the potential benefits of incorporating a specially prepared cell wall, which has not been investigated previously (*30*, *31*). We expect the protective role of a cell wall to be more pronounced especially when facing the osmotic pressure under the complex gastrointestinal (GI) system due to the remarkable stiffness and strength of cellulose nanofibers (CNFs). In addition, cellulose is a type of dietary fibers, which can promote intestinal peristalsis, repair the intestinal barrier, and improve the composition of the gut microbiota (*32*, *33*).

**Fig. 1.**
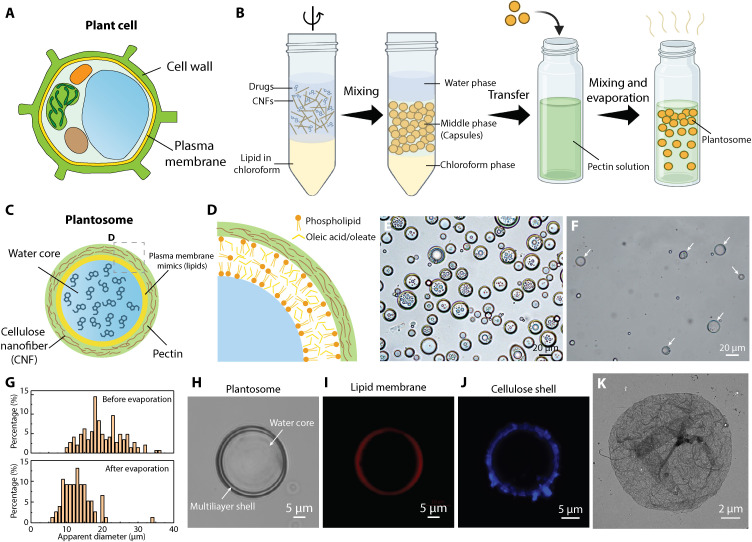
Fabrication and characterization of plantosomes. (**A**) Illustration of a plant cell with its cell wall and plasma membrane. (**B**) Preparation protocol of cargo-loaded plantosomes. (**C**) Simplified illustrations of a cargo-loaded plantosome. (**D**) Close-up of the proposed CNF/pectin and lipid layer structure. (**E** and **F**) Bright-field images of suspensions before (E) and after (F) the evaporation of chloroform and coating with pectin. The white arrows point to plantosomes. (**G**) Statistics of the size distribution of plantosomes before and after evaporation. (**H**) Bright-field image of a single plantosome. (**I**) CLSM image of a plantosome with Rh-DOPE (red) in the lipid phase. (**J**) CLSM image of a plantosome showing CNFs stained with calcofluor-white (blue). (**K**) TEM image of the fibrous cellulosic shell. Figure created with BioRender.com.

The aim of this study was to assemble synthetic plant cells, termed plantosomes, using CNFs, pectin, oleic acid (OA), and phospholipids, and to demonstrate their oral drug delivery potential by studying their release properties in simulated gastrointestinal and intestinal fluids. In addition, we evaluated whether the plantosomes had any toxicological effects on an intestinal cell line. The plantosomes mimic the structure in plant cells, consisting of a cell wall construction (CNF/pectin shell) encasing a plasma membrane mimic (lipid layer) with a water core (Fig. 1). We selected several different fluorescein isothiocyanate (FITC)–dextrans as model drugs and demonstrated that plantosomes can encapsulate cargos of various sizes (average *M*_w_ from 4 to 2000 kDa, which correspond to hydrodynamic diameters of ~2 to ~200 nm). The present system is also suitable for loading other types of cargo, as evidenced by the successful encapsulation of 24-nm polystyrene nanoparticles. The importance of the lipid layer for encapsulation and release was evaluated by comparing plantosomes with carriers consisting only of a CNF/pectin shell, i.e., without a lipid layer. The structure of plantosomes was altered based on the stimuli-responsive behavior of the lipid layer, causing a change in permeability properties. This endows the system with dual-triggered release mechanisms with the potential to deliver multiple cargos in response to external stimuli at specific target sites in the GI tract.

## RESULTS

### Fabrication and characterization of plantosomes

Plant cells (Fig. 1A) are complex multicompartment structures containing a large variety of plant organelles, each of which fulfills specific cellular functions. To support these interior functionalities, an outer shell, composed of a cell wall and plasma membrane, provide mechanical support and regulate the passage of nutrients to and from the plant cell’s interior. The primary cell wall of a living cell is composed of pectins, hemicelluloses, and cellulose microfibrils, whereas the inner plasma membrane consists of lipids (glycerolipids, sphingolipids, and phytosterols) (*34*) and transmembrane proteins (*35*), creating a selectively permeable barrier that controls molecular passage. The limiting pore diameter in real cell walls depends on the plant source and is estimated to be 3.5 to 9.2 nm (*6*). Hence, molecules of such sizes or larger are expected to be entrapped by the cell wall only. In addition, pectin is thought to be the primary determinant of wall porosity (*6*). The combination of a cell wall and a plasma membrane makes the plant cell an excellent natural container for cargo delivery.

To mimic the structure of the outer barrier in plant cells, we combined an emulsification step with a coating technique to self-assemble plantosomes (Fig. 1B). The CNF suspension (fig. S1), lipids (phospholipids and OA) in chloroform, and a model compound as cargo were emulsified using a short and pulsed high-speed mixing protocol. After settling, the emulsion in the middle phase was transferred for further coating with pectin followed by chloroform evaporation. Notably, no stable emulsion could be obtained without CNFs at the present processing pH range (*2*, *36*). The positively charged CNFs, combined with lipids, stabilize the water/oil interface of the formed lipid layer/oil droplets. The amphiphilic and Pickering stabilizing properties of CNFs, used to stabilize oil droplets in water, have been extensively investigated in the past (*30*, *37*–*39*). In addition, the adsorption of anionic sugar beet pectin was facilitated by ionic interactions with cationic CNFs (fig. S2). As a result, plantosomes exhibit a structure with a CNF-reinforced pectin shell and a lipid layer encasing a large water core (Fig. 1C). As shown in Fig. 1D, the lipid layer comprises OA/oleate and a small amount of phospholipids. It should be noted that the fabrication protocol inadvertently produced a mixture of plantosomes and capsules, with capsules containing either only a lipid core or, in some cases, smaller water droplets (Fig. 1F). This variation can be attributed to the production protocol, as shown in Fig. 1B. Here, we optimized the ratio between phospholipids and OA to obtain a large population of plantosomes (fig. S3). Both the internal water and lipid phase can carry cargo, but in the present study, the focus was on encapsulating hydrophilic molecules into the water core. Mimicking the structure represents an important step toward synthetic plant cells, especially for encapsulation applications (*2*). Before chloroform evaporation and coating, the droplet size in suspension was 19.9 ± 5.3 μm (Fig. 1, E and G), whereas the final plantosomes had an average diameter of 12.3 ± 2.8 μm (Fig. 1, F and G). A low CNF concentration (0.059 wt %) was selected to limit the amounts of CNFs entering the interior water core of plantosomes and to attain sizes similar to that of real plant cells (10 to 100 μm). A higher CNF concentration leads to smaller droplet sizes of 6.0 ± 2.1 μm (fig. S4). To identify the CNF shell around the plantosomes (Fig. 1H), cellulose was stained with calcofluor-white (Fig. 1J) and Carbotrace 630 (fig. S5). Pectin was visualized after lipid removal by staining the pectin with propidium iodide (PI) (fig. S6). Moreover, the presence of CNFs was further shown by transmission electron microscopy (TEM) imaging of the shell after lipid removal (Fig. 1K), where the fibrous shell structure was observed fig. S7. The position of the lipid layer was found by introducing a fluorescently labeled phospholipid Rh-DOPE [1,2-dioleoyl-*sn*-glycero-3-phosphoethanolamine-*N*-(lissamine rhodamine B sulfonyl)] in the lipid layer (fig. S8). As indicated in Fig. 1I, a thin lipid layer was present in plantosomes. Colocalization CLSM (confocal laser scanning microscopy) images of the lipid layer (Rh-DOPE, red) and cellulose shell (CFW, turquoise) are shown in fig. S9. Although cellulose has been extensively used in drug delivery systems in various forms such as micro/nanoparticles (*40*), films (*41*), and hydrogels (*42*), this marks the first instance of embedding CNFs with pectin, then integrating a lipid barrier, to engineer a delivery system inspired by the plant cell wall and plasma membrane structure.

### Encapsulation of cargoes with different sizes

**Fig. 2.**
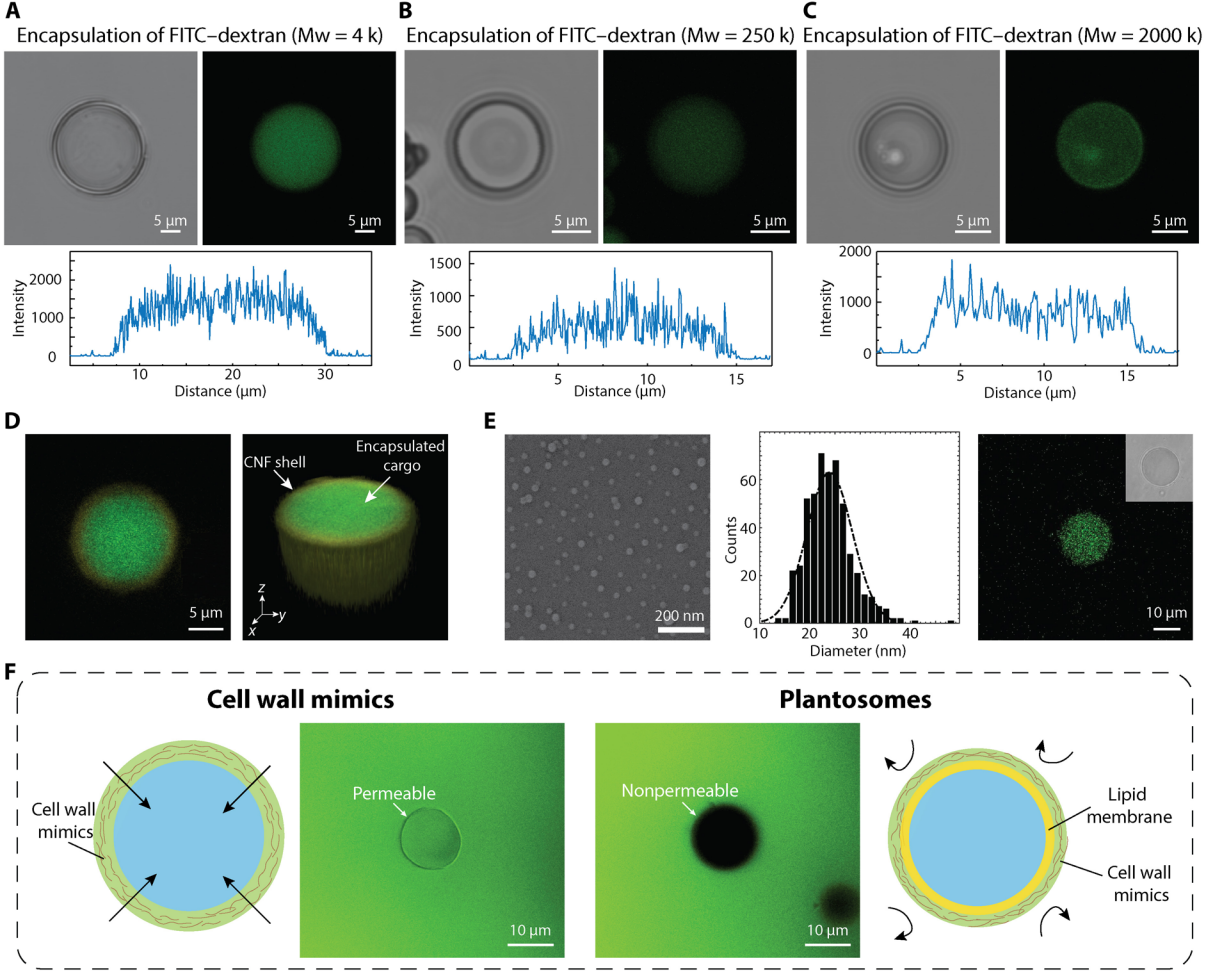
Encapsulation of different FITC-dextrans in plantosomes. (**A** to **C**) Bright-field images, CLSM images, and fluorescence intensity of plantosome with encapsulated 4-kDa FITC-dextran (green) (A), 250-kDa FITC-dextran (B), and 2000-kDa FITC-dextran (C), respectively. (**D**) 3D reconstructed CLSM image of the encapsulated core in plantosomes [Carbotrace 680 (yellow) and 4-kDa FITC-dextran (green)]. (**E**) TEM image, size distribution, and encapsulation of fluorescent latex beads (polystyrene nanoparticles). Inset: Bright-field image of the plantosome. (**F**) Comparison of permeability test results of cell wall mimics (without lipid layer) (left) and blank plantosomes (right) in the presence of 4-kDa FITC-dextran (green, 0.4 mg/ml) and illustrations of cell wall mimics and plantosomes.

To demonstrate the encapsulation capability of plantosomes, FITC-labeled dextran with three different sizes (4, 250, and 2000 kDa) were selected (*43*). FITC-dextran was added to the water phase (fig. S10) due to the good water solubility of these compounds. After encapsulation, free FITC-dextran was removed by dialysis. Bright-field microscopy and corresponding CLSM images (Fig. 2A) showed that the fluorescent molecules (4-kDa FITC-dextran) were successfully encapsulated in the interior water core of the plantosomes. Similar trends were observed for 250-kDa FITC-dextran and 2000-kDa FITC-dextran in Fig. 2 (B and C), respectively. The even distribution of FITC-dextran across the water core (green) and cellulose shell stained with Carbotrace 680 (yellow) was visible in the reconstructed three-dimensional (3D) images (Fig. 2D). Notably, FITC-dextran was present in the interior of the plantosomes even after extensive dialysis of 7 days (fig. S11), further demonstrating the excellent encapsulation capacity for a wide range of sizes and showing notable potential for delivering different drugs and compounds. Evidently, the lipid layer creates a barrier that enhances encapsulation ability (*44*, *45*). The encapsulation efficiency for 4- and 2000-kDa FITC-dextran was 21.5 ± 4.3 and 12.6 ± 4.6%, respectively (figs. S12 to S14). The efficiency is related to the volume fraction of internal water, thus increasing the water fraction in the interior is expected to enhance the encapsulation efficiency. In addition, we note that the amount of plantosomes will vary between different between batches, which influences the encapsulation efficiency. Any residual cargo in the water (top) phase (fig. S10B) could be collected for recycling. It is worth mentioning that plantosomes were compatible with loading other molecules. As a support of this, fluorescently labeled latex beads (polystyrene nanoparticles) with an average size of 24 nm were successfully encapsulated (Fig. 2E).

The importance of the lipid layer for trapping and retaining cargoes of different sizes was further supported by comparing the permeability of blank plantosomes (without cargo loading) with layer-by-layer (LbL) capsules, also referred to as cell wall mimics, which only consisted of a CNF/pectin shell. The cell wall mimics were prepared by assembling CNFs and pectin with the LbL adsorption protocol (*46*). As shown in Fig. 2F, 4-kDa FITC-dextrans easily permeated through the shell of the LbL capsule but not through the shell of the plantosome. The shell in the LbL capsules composed of six bilayers of CNF/pectin, which, despite its thickness, remained porous enough to allow passage of 4-kDa FITC-dextran. Therefore, the higher barrier property of the plantosomes was attributed to the lipid layer. The findings align with the role of natural plant cell walls, where the plasma membrane regulates the passage of nutrients. Increasing the phospholipid amount is expected to increase the barrier properties further (*47*). The fluidity and permeability properties of the lipid layer typically increases with OA inclusion (*48*–*50*). We also note that the high proportion of OA in the lipid layer, coupled with the presence of CNFs, suggests that our system could potentially be used for probiotic delivery. OA has been shown to enhance the survival rate of probiotic lactobacilli in gastric juice (*51*), whereas cellulose has been successfully used as a carrier for probiotic delivery, promoting the survival and colonization of loaded probiotics (*32*).

### Stability test of plantosomes in vitro

**Fig. 3.**
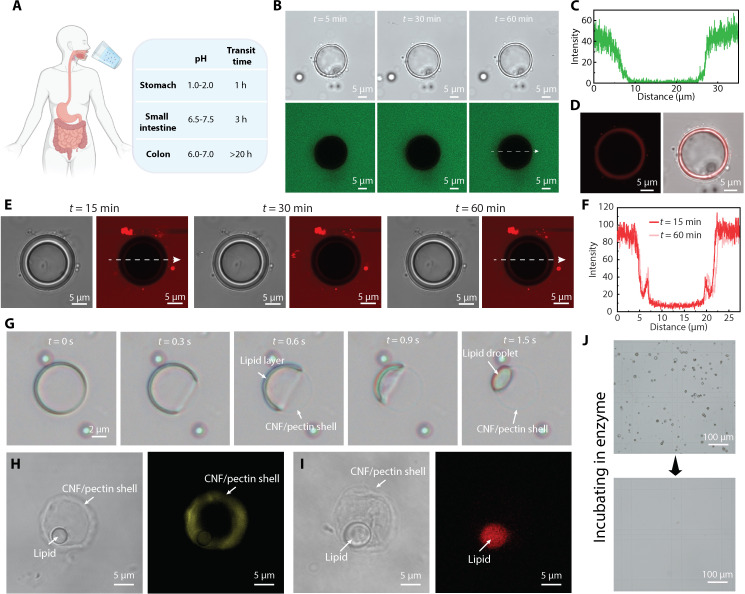
Stability test of plantosomes in biorelevant media. (**A**) Scheme of the GI tract in the human body. h, hours. (**B**) Stability test of plantosomes in FaSSGF in the presence of 10-kDa pHrodo-dextran (0.4 mg/ml; green). (**C**) Fluorescence intensity of the pHrodo (green) along the arrow line in (B) after 60-min incubation. (**D**) CLSM image and bright-field image of the same plantosome as in (B) where the lipid layer was stained with Rh-6G. Rh-6G staining was performed after 1 hour of incubation in the FaSSGF. (**E**) Bright-field and CLSM images of a plantosome incubated in FaSSGF in the presence of pHrodo-labeled pepsin (0.4 mg/ml; red) for 1 hour. (**F**) Corresponding fluorescence intensity curve at 15 and 60 min. (**G**) Stability test of plantosomes in FaSSIF. (**H** and **I**) Bright-field image and CLSM image of a plantosome, showing the CNF shell stained with Carbotrace 630 (yellow) (H) and the lipid droplet containing Rh-DOPE (red) (I) after the lipid layer shrinkage in FaSSIF. (**J**) Bright-field images of plantosomes in a Bürker chamber before (top) and after (bottom) exposure to pectinase and cellulase. Figure created with BioRender.com.

An in vitro study was conducted using biorelevant media and enzymes simulating the environments in the GI tract to evaluate the potential of plantosomes for site-specific delivery (*52*, *53*). The GI tract is an intricate system composed of different specialized organs with distinct chemical environments and varying residence times (Fig. 3A) (*22*, *54*). To replicate the physiological conditions and residence time in the stomach, the plantosomes were incubated in fasted state simulated gastric fluid (FaSSGF) at a pH of 1.78 for 1 hour. According to the bright-field image sequence (fig. S15), the plantosome exhibited no damage under gastric conditions. Furthermore, 10-kDa pHrodo-dextran (green) was introduced in the FaSSGF to simulate the enzymes in stomach, and the permeability was tested at low pH. According to the CLSM image sequence (Fig. 3B), the lipid layer is able to block out the 10-kDa molecule after 1-hour incubation as nearly zero signal was be found inside the plantosomes (Fig. 3C). The plantosome exhibited no damage under gastric conditions. The lipid layer was stained with rhodamine 6G (Rh-6G, red) in the end of the experiment (Fig. 3D). OA is pH sensitive (*36*) and forms an oil-in-water emulsion at low pH (<7), with other phases (cubic, lamellar, and micellar phases) found at increasing pH values above 7 (*55*). Here, the lipid layer of plantosome was a combination of OA and phospholipid and it remained stable under the harsh acidic conditions. Moreover, the size of the plantosome increased slightly from 20.0 to 21.5 μm, likely due to the difference in salt concentration inside (0.1 M) and outside (0.06 M) the plantosome, leading to a net influx of water into the plantosome and subsequent expansion. We note that, because of the present composition of the lipid layer, which is static and not growing by the inclusion of more OA, it is not possible to maintain a pH gradient between the interior and exterior (*56*). However, the pH in the stomach can be increased to as much as pH 6, with food intake (*57*). To further assess whether the current lipid layer can effectively block pepsins in the stomach, we incubated plantosomes with pHrodo-labeled pepsin (red) (Fig. 3E). The results demonstrate that the lipid layer prevented pepsin from entering, and the fluorescence signal in the interior remained largely unchanged at 15 and 60 min (Fig. 3F). The weak signal in the interior is likely due to the unreacted and free pHrodo, which subsequently adsorbed to the lipid layer. Additional evidence of free pHrodo dye (red) penetrating the lipid layer of plantosome is provided in fig. S16. Overall, the lipid layer and pectin/CNF wall of the plantosomes provided protection for the encapsulated cargo from the enzymes in FaSSGF.

By changing the medium from FaSSGF to FaSSIF (fasted state simulated intestinal fluid), the stability of plantosomes in the small intestine was investigated. FaSSIF contains bile salts and phospholipids and has a pH of 6.5. The plantosomes exhibited structural changes within a few seconds after incubating in FaSSIF (Fig. 3G and movie S1). We hypothesize that this process is caused by the shrinkage of the lipid layer and the formation of a lipid droplet, which was observed in the corner of plantosome after *t* = 1.5 s. The remaining “ghost” ring was the CNF/pectin shell. Carbotrace 630 (yellow) and Rh-DOPE (red) were introduced to identify the different parts. As indicated in CLSM micrographs in Fig. 3 (H and I), the retained ring was a CNF/pectin shell, and it had buckled to some extent as the interior lipids pulled away from the shell. The red-stained droplet originated from the shrinkage of the lipid layer. We note that the lipid droplets are emulsified in FaSSIF and eventually disappear from the interior of the remaining CNF/pectin shell (fig. S17). Thus, the structure of the plantosome was morphed based on the stimuli-responsive behavior of the lipid layer. Bile salts are produced by the liver and secreted into the small intestine where the bile salts solubilize and emulsify lipids (*22*). This behavior largely hinders the application of drug delivery systems based on liposomes for targeting remote parts of the GI tract, e.g., the large intestine, as they disintegrate in the small intestine. For example, self-nanoemulsifying drug delivery systems (SNEDDS), which represents current advanced drug delivery system, spontaneously forms fine oil-in-water nanoemulsions to enhance bioavailability but can only achieve the targeted delivery in the small intestine (*58*). On the other hand, for our bioinspired system, the CNF/pectin shell of plantosomes (fig. S18) provided further protection after passing through the small intestine and could therefore potentially be used for targeted drug release to the colon.

The remotest part of GI tract, the colon, is populated by a variety of gut bacteria that secrete enzymes (e.g., glucosidase and pectinase) for polysaccharide degradation (*13*, *59*). This provides the potential to break down the plant cell wall (including microcrystalline cellulose) (*59*) for targeted drug release to the colon (*11*, *12*). Here, we used two polysaccharidases, cellulase and pectinase, to imitate the microbial environment of the colon in vitro. As shown in Fig. 3J, after incubation in a buffer with these enzymes for 2 days, the population of plantosomes was degraded, indicating the possibility of breaking the synthetic cell wall of plantosomes in the colon. Thus, a two-step decomposition of plantosomes was realized. First, the lipid layer shrank due to the bile salts, and then the CNF/pectin shell was degraded in the presence of enzymes.

### In situ observation of the size-dependent release mechanism in FaSSIF

The shrinkage of the lipid layer in FaSSIF and its impact on the encapsulation properties of plantosomes was further investigated. Plantosomes loaded with 4- and 2000-kDa FITC-dextran were incubated in FaSSIF and observed in situ under CLSM. The plantosome shells were stained with Carbotrace 680 (yellow) for visualization.

**Fig. 4.**
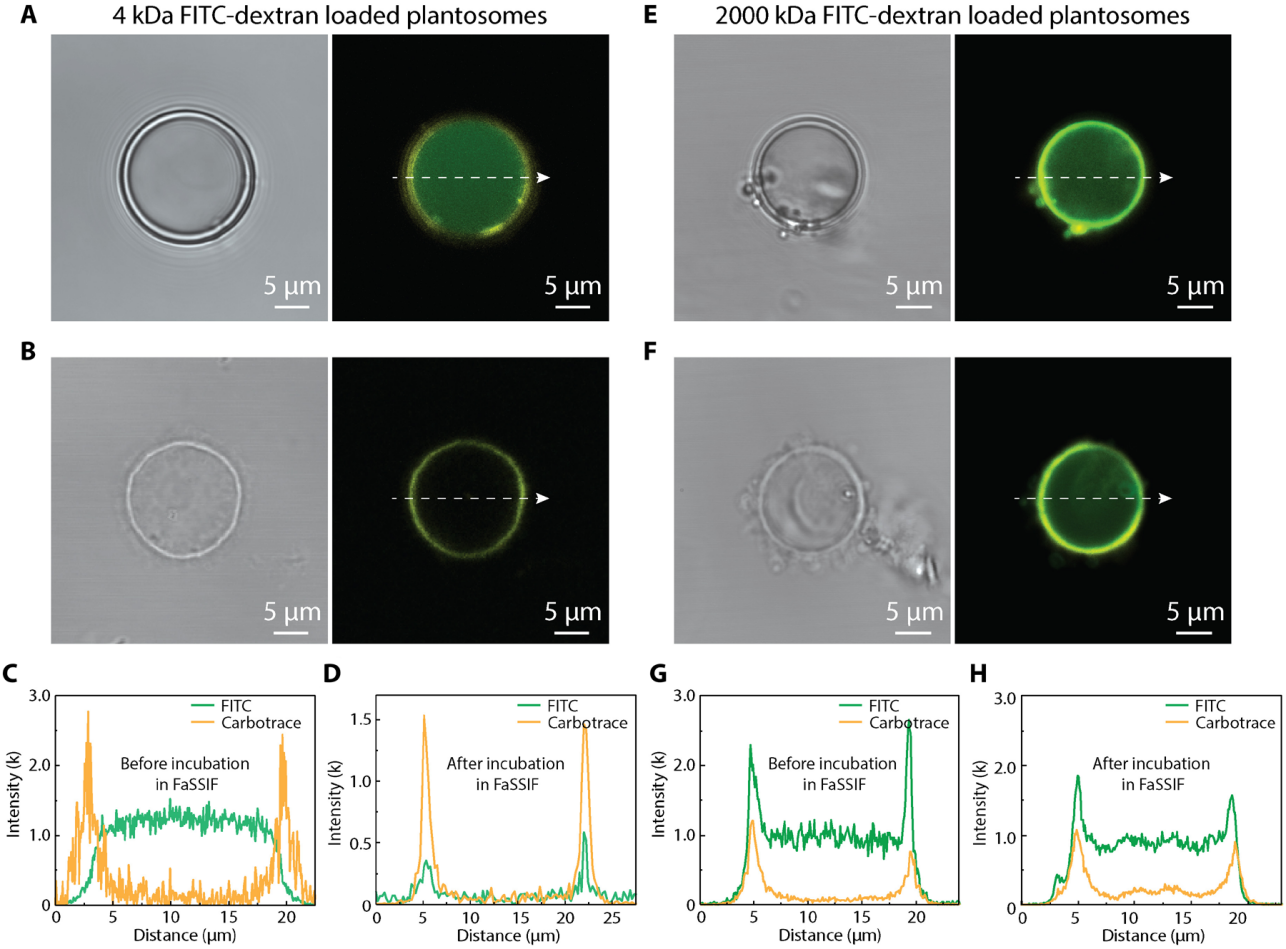
Influence of cargo size on the release mechanism in FaSSIF. (**A**) Bright-field image and CLSM image of a plantosome loaded with 4-kDa FITC-dextran in 0.1 M NaCl. (**B**) Bright-field image and CLSM image of 4-kDa FITC-dextran–loaded plantosome after incubation in FaSSIF. (**C** and **D**) Fluorescence intensity of FITC (green) and Carbotrace channel (yellow, Carbotrace 680) for 4-kDa FITC-dextran–loaded plantosomes before (C) and after (D) incubation in FaSSIF. (**E**) Bright-field image and CLSM image of a plantosome loaded with 2000-kDa FITC-dextran in 0.1 M NaCl. (**F**) Bright-field image and CLSM image of 2000-kDa FITC-dextran–loaded plantosome after incubation in FaSSIF. (**G** and **H**) Fluorescence intensity of FITC (green) and Carbotrace channel (yellow, Carbotrace 680) for 2000-kDa FITC-dextran–loaded plantosomes before (G) and after (H) incubation in FaSSIF.

At first, plantosomes loaded with 4-kDa FITC-dextran displayed a strong fluorescent signal (green) in the water core (Fig. 4A). After incubation in FaSSIF and subsequent removal of the lipid layer, the interior fluorescent signal disappeared, and the CNF/pectin shell became clearly visible (Fig. 4B). The quantified fluorescent intensity of FITC shows high level before incubation in FaSSIF (Fig. 4C). The disappearance of the fluorescent signal of the core (Fig. 4D) indicates the complete release of the cargo after the loss of the lipid barrier. This is accompanied by the emergence of a distinct fluorescent signal of the shell (Fig. 4D).

In the plantosomes loaded with 2000-kDa FITC-dextran, the cargo was distributed in the water core with some additional dextran also being adsorbed onto shell due to electrostatic interactions (Fig. 4E) and the present assembly protocol (Fig. 1B). After lipid removal, the more than 60 times larger molecules {with respect to their average hydrodynamic diameter of 173.9 nm [dynamic light scattering (DLS)], compared to 2.7 nm for 4-kDa FITC-dextran}, mainly remained within the CNF/pectin shell (Fig. 4F). The fluorescence intensity before and after lipid removal further supported this finding (Fig. 4, G and H). Apart from these, FITC-dextrans with other sizes were tested. The results (fig. S19) show that the 70-kDa FITC-dextrans were released in the absence of the lipid layer barrier. The 150-kDa FITC-dextran population was partly released from the CNF/pectin shell, with a notable amount of them sticking on the shell. However, the 250-kDa FITC-dextrans were largely retained after the lipid removal. As the limiting diameter in real cell walls is estimated to be 3.5 to 9.2 nm (*6*), there is room for further improvement of the present plantosome systems. We hypothesize that reducing the sensitivity of the pectin/CNF polyelectrolyte complex in the wall to NaCl (*60*, *61*), e.g., by exploiting cross-linking strategies that are found in real plant cell walls (*7*, *62*, *63*), could further entrap and deliver molecules with hydrodynamic diameters of smaller sizes (less than ≈150 kDa) to the colon. An intact CNF/pectin shell is thus important for containing larger entities all the way to the colon. In contrast, the lipid layer is essential for entrapping smaller molecules that are released in the small intestine.

### Dual-triggered release mechanism

**Fig. 5.**
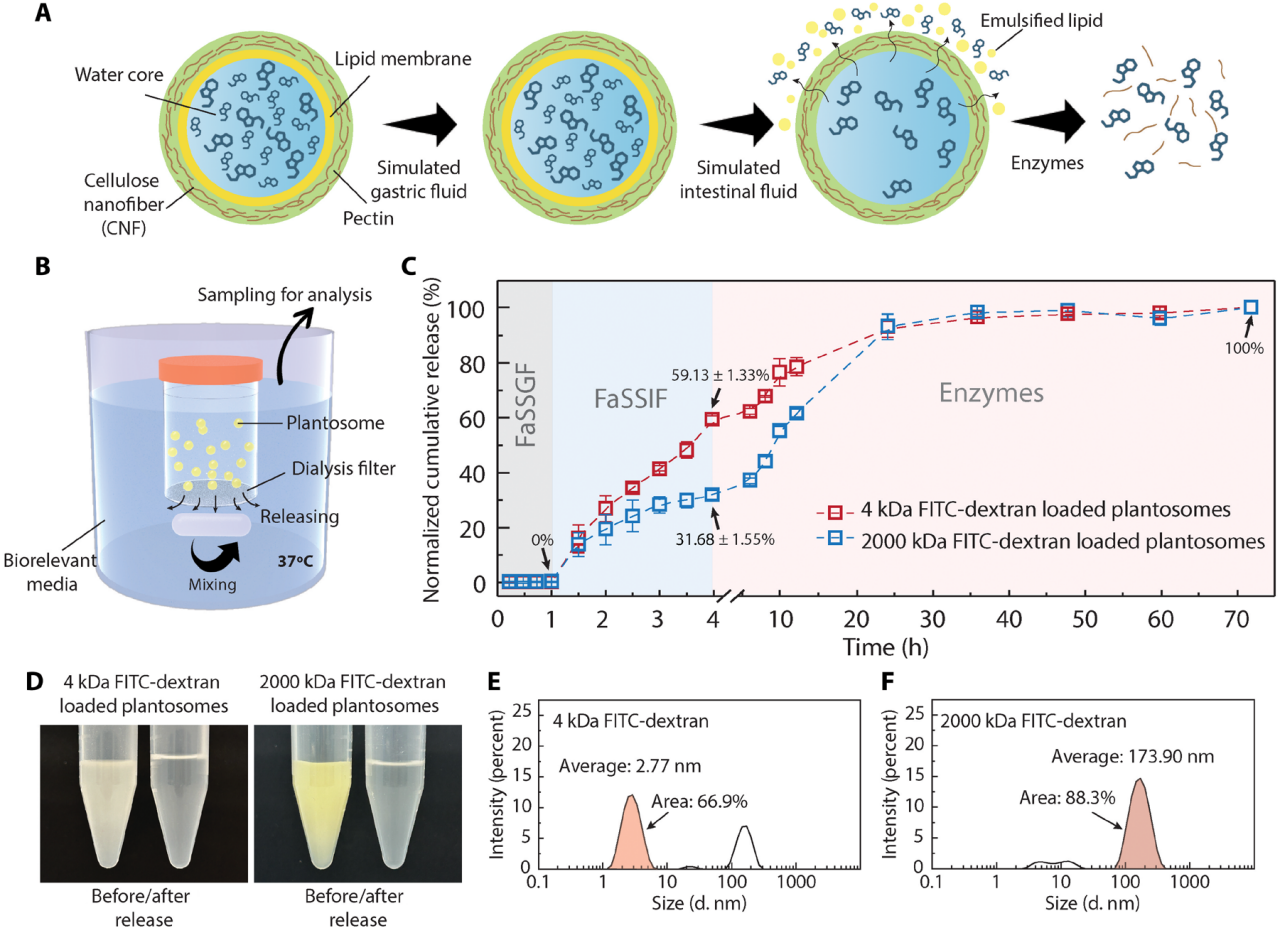
Dual-triggered release mechanisms in plantosomes. (**A**) Scheme of the two-step release mechanism depending on cargo size. (**B**) Illustration of the device for the in vitro release study. (**C**) Sequential release curves of plantosomes loaded with 4- or 2000-kDa FITC-dextran. (**D**) Photos of FITC-dextran–loaded plantosomes before/after release study. DLS measurement results showing the size distribution of 4-kDa (**E**) and 2000-kDa (**F**) FITC-dextran.

The schematic in Fig. 5A summarizes the dual-triggered release mechanism, we have reported so far, for plantosomes. To further quantify the two-step triggered release behavior in vitro, especially regarding size selectivity, we conducted a sequential release study. The illustration of the device is shown in Fig. 5B. Plantosomes with cargoes were placed in a container with a 5-μm filter at the bottom to allow rapid substance exchange. The release study was conducted stepwise by placing the container first in FaSSGF, then FaSSIF, and lastly in a buffer (pH = 5) with enzymes at 37°C. Aliquots were sampled at specific time intervals, and the cumulated release data were calculated (figs. S20 and S21). The release behavior of plantosomes loaded with either 4- or 2000-kDa FITC-dextran was compared.

In the first hour, no FITC-dextran, irrespective of size, was released in simulated gastric fluid (Fig. 5C). After transfer to FaSSIF, plantosomes loaded with small molecules (4 kDa) exhibited a fast release, reaching 59 ± 1.3% after 3 hours. Notably, the slope of the release curve (red) did not reach a plateau at *t* = 4 hours, indicating that the release step was not yet completed. In contrast, plantosomes loaded with the 2000-kDa FITC-dextran molecules showed only a release of 32 ± 1.6% (blue curve) after incubation in FaSSIF, with the slope flattening at *t* = 4 hours. The 30% difference in release ratio in FaSSIF indicated selective release at different sites according to cargo size, supporting our assumption in Fig. 5A. Furthermore, nearly 100% release was achieved for both cargo sizes after incubation in a buffer with enzymes for 1 day.

Visual inspection (photos in Fig. 5D) of the cargo-loaded plantosomes before and after the release study showed increased transparency and a noticeable color change, indicating breakage of the plantosomes and release of cargo. DLS analysis revealed that both FITC-dextrans contained a range of dextran sizes; only 66.9% of the 4-kDa molecules had a size distribution around the hydrodynamic diameter of 2.77 nm (Fig. 5E), and 88.3% of the 2000-kDa molecules were approximately the hydrodynamic diameter of 173.9 nm (Fig. 5F). Taking this into account in the normalization, the selectivity of 4-kDa molecules released by FaSSIF reached 88.4%, and 2000-kDa molecules released by enzyme reached 77.4%. The high selectivity offers great potential for multitargeted delivery of drugs.

### Cytotoxic properties of plantosomes

**Fig. 6.**
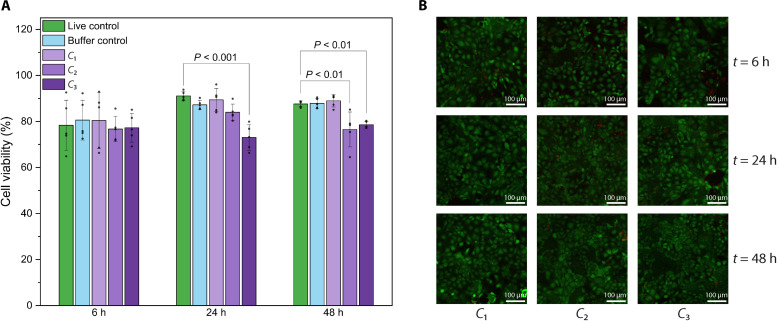
Cytotoxicity test of plantosomes. (**A**) Viability of Caco-2 cells after 6, 24, and 48 hours of incubation with media (live control), blank solution (buffer control), and different concentrations of plantosomes (*C*_1_, *C*_2_, and *C*_3_) calculated from LDH leakage data (*n* = 5, means ± SD). A one-way ANOVA with Tukey’s post hoc test was performed to determine the statistical significance versus the live control and is given as *P*. (**B**) Fluorescent confocal microscope images of LIVE/DEAD-stained Caco-2 cells after 6, 24, and 48 hours of incubation with three different concentrations of plantosomes (*C*_1_, *C*_2_, and *C*_3_). Live cells (green) were stained with Calcein AM, whereas dead cells (red) were stained with BOBO-3 Iodide.

Plantosomes are inspired by the intricate architecture of plant cells and are constructed using components that minor their natural counterparts. This biomimetic approach leverages the nontoxic nature of plant constituents, including fatty acids, phospholipids, and cell wall components like cellulose and pectin. The pectin used for plantosomes was extracted from sugar beet pulp, whereas CNFs were derived from wood pulp. Here, the CNFs were chemically modified to contain positively charged functional groups (0.6 mmol g^−1^) on their surfaces. This modification facilitates electrostatic interactions with pectin, which carries negatively charged deprotonated carboxylic acid groups at neutral or basic pH. Previous studies have investigated CNFs with different surface modifications, including positive charges, and have found no signs of cytotoxicity (*64*, *65*). The safety of the bioinspired cargo delivery system was investigated further, focusing on the colon as its target organ. Three different concentrations of plantosomes (*C*_1_ = 20%, *C*_2_ = 100%, and *C*_3_ = 200%) were tested with Caco-2 cells (human colorectal adenocarcinoma cells) over 6, 24, and 48 hours. The biocompatibility was evaluated using two assays, the lactate dehydrogenase (LDH) assay to assess cytotoxicity and the live/dead assay to visualize cell viability directly. The calculated cell viability from LDH leakage data is shown in Fig. 6A. After 6-hour treatment, no substantial decrease in cell viability was found compared to the live control. However, the overall mean cell viability after 6-hour exposure was lower than after 24 and 48 hours. This can be attributed to the cells not having reached full confluency at the start of the treatment, which left their cell membranes more vulnerable (*66*, *67*). In addition, exposure of the nanoparticles to the cells may cause initial stress and reduce the viability in a short time. The increased susceptibility could be linked to higher LDH leakage, an indicator of cell membrane integrity, and consequently lower overall cell viability. By concentration *C*_1_ and buffer control, no membrane-damaging effects could be found for all three time points tested. Treatment with *C*_2_ led to a decrease in cell viability after 48 hours (*P* < 0.01). Also, treatment with the highest dose, *C*_3_, resulted in a reduction after 24 hours (*P* < 0.001) and 48 hours (*P* < 0.01), revealing dose- and time-dependent effects. Still, the treatment had minimal cytotoxic effects, and the cells remained viable. The findings were corroborated by the fluorescent confocal microscope images of LIVE/DEAD-stained Caco-2 cells (Fig. 6B). An increase in cell density was observed with time, resulting in a greater number of densely packed living green fluorescent cells after 24- and 48-hour incubation with plantosomes. The images showed that the cells were essentially unchanged for all three concentrations. Even after a further increase in the incubation time to 48 hours, no decrease in viability could be observed, indicating excellent biocompatibility of the plantosomes. The absence of cytotoxic effects for a long period is essential for a potential application in colon-targeted oral drug delivery, which can have a delayed transit time of up to 48 hours (*68*).

## DISCUSSION

In conclusion, we developed a type of cargo delivery system inspired by the structure of plant cells. Plantosomes, featuring cell wall and plasma membrane mimics, show the encapsulation capability of a range of cargo sizes and exhibit resistance to acidic conditions in simulated gastric fluid. The lipid layer shows a stimuli-responsive behavior due to bile salts present in the GI fluids, resulting in an instant release of small molecular cargoes (4- and 70-kDa FITC-dextrans) to the simulated intestinal fluid. Yet, the structurally stable cellulose/pectin shell further ensures the delivery of large cargoes (here, 150-, 250-, and 2000-kDa FITC-dextran tested) to a simulated colonic fluid, where enzymes ultimately degrade the shell. This size-dependent triggered release mechanism of two payloads shows the potential in releasing small and larger cargoes at different sites in the GI tract. Moreover, the plantosomes show excellent biocompatibility when incubated with Caco-2 cells.

This work opens exploration pathways for developing the next generation of bioinspired multisite release systems, targeting combination therapies and coordinated body responses. Given the mechanically robust structure of the synthetic cell wall in plantosomes, in combination with the lipid properties, the present system holds great potential of surpassing the small intestine and delivering macromolecules, probiotics, proteins, and nanoparticles to colon in the future, thereby addressing existing challenges with conventional oral drug delivery systems based on lipids alone.

## MATERIALS AND METHODS

### Materials

CNFs modified with cationic groups (trimethylammonium groups) were prepared according to the previous protocol (*69*). The amount of cationic groups was 0.6 mmol g^−1^ CNF attained with conductimetric titration (*70*). The GENU BETA pectin, a high ester pectin extracted from sugar beet pulp, was a kind gift from CP Kelco (Lille Skensved, Denmark). Sodium chloride, sodium hydroxide, hydrochloric acid, sodium taurocholate, sodium phosphate, sodium citrate tribasic dihydrate, chloroform, OA, POPC (2-oleoyl-1-palmitoyl-*sn*-glycero-3-phosphocholine), POPE (2-oleoyl-1-palmitoyl-*sn*-glycero-3-phosphoethanolamine), FITC-dextran (with 4, 70, 150, 250, and 2000 kDa), calcofluor-white stain, pectinase from *Aspergillus aculeatus* (aqueous solution, ≥800 U/ml), and cellulase from *Trichoderma* sp. (powder, ≥5000 U/g solid) were purchased from Sigma-Aldrich (Sweden). Rh-DOPE (ammonium salt) was purchased from Avanti Polar (USA). Rh-6G, latex beads (aqueous suspension, 0.03-μm mean particle size), carboxylate-modified polystyrene, fluorescent yellow-green were purchased from Sigma-Aldrich (Sweden). FaSSGF and FaSSIF were purchased from Biorelevant (UK). Carbotrace 630 and 680 were kind gifts from Ebba Biotech (Sweden). pHrodo Green Dextran (10,000 MW) and pHrodo iFL red STP ester (amine-reactive dye) were purchased from Thermo Fisher Scientific. Pepsin from porcine gastric mucosa (lyophilized powder, ≥3200 U mg^−1^) was purchased from Sigma-Aldrich (Sweden). The pepsin was labeled with pHrodo iFL red STP ester at a dye:protein molar ratio of 9:1 following the manufacturer’s protocol, with the exception that the reaction took place (1 hour) at pH 7. The labeled pepsin was purified with dialysis (first against Milli-Q with pH 3, then FaSSGF).

### Preparation of stock solutions

The concentrated CNFs were diluted to 0.059 wt % with Milli-Q water in the presence of 0.1 M NaCl, mixed overnight, and then sonicated under 70% amplitude for 1 min. Pectin solution with 0.2 wt % concentration was prepared by dissolving 100 mg of pectin powder in Milli-Q water in the presence of 180 μl of 1 M NaOH. The pH of above solutions was adjusted to 7 ± 0.2 before use. The 288 mM OA in chloroform solution was prepared by mixing 0.94 g of OA in 15.64 g of chloroform and stored at 4°C. The POPC/POPE in chloroform stock solution was prepared in concentrations of 1 and 0.2 mM, respectively, and stored at −20°C. Rh-DOPE/POPC/POPE in chloroform solution with the respective concentrations of 0.09, 1, and 0.14 mM was prepared by mixing fluorescently labeled phospholipid (Rh-DOPE) with POPC and POPE and stored at −20°C.

### Atomic force microscopy

CNF (0.001 wt %) was dispersed in water and stirred overnight (350 rpm) at room temperature and then sonicated under 70% amplitude for 1 min. The silicon wafer was washed three times with water and ethanol, followed by plasma treatment. Twenty-five microliters of CNF solution was dropped on the silicon wafer and incubated for 1 min and then dried under nitrogen gas flow. Atomic force microscopy images were obtained by a Bruker NanoScope V (USA).

### Preparation of plantosomes

For the preparation of blank plantosomes, 2.3 g of 0.059 wt % CNF in 100 mM NaCl suspension, 1 ml of 288 mM OA solution, 0.5 ml of chloroform, and 0.5 ml of POPC/POPE solution were mixed three times under 24k rpm (15 s each time). The low amount of CNF, amphiphilic properties of CNFs, pulsed mixing, and NaCl minimizes CNF entering the water core. After settling down for 30 min, 0.3 ml of the CNF stabilized droplets in the middle phase was transferred to 10 ml of 200 mM NaCl solution and sucked and released for several times with a pipette. Then, 10 ml of 0.2 wt % of pectin solution was added, and the obtained mixture was stirred (350 rpm) overnight to allow the evaporation of chloroform and coating of pectin. Then, the suspension with plantosomes (pH ≈ 5.1) was obtained. The percentage of plantosomes was 20.8 ± 4.9%.

For the preparation of FITC-dextran–loaded plantosomes, 2.7 mg of FITC-dextran powder was added separately before the first mixing step. The lipid-labeled plantosomes were prepared by changing the initial formulation before mixing, as listed below: 2.3 g of 0.059 wt % CNF in 100 mM NaCl suspension, 1 ml of 288 mM OA solution, 0.5 ml of chloroform, 0.48 ml of the POPC/POPE stock solution, and 40 μl of the Rh-DOPE/POPC/POPE phospholipid stock solution. The plantosomes were dialyzed with 0.1 M NaCl solution by a 5-μm filter for 2 days prior to use. The preparation of cell wall mimics for comparison was based on the LbL technique; details of the preparation protocol can be found in ref. (*46*).

### Calculation of encapsulation efficiency

The standard curve between different concentrations of FITC-dextran and fluorescence intensity was tested first. Then, the accumulated fluorescence intensity of released FITC-dextran in the release study was tested, and the concentration was determined based on the standard curve. Thus, the amount of the release FITC-dextran was obtained. The encapsulation efficiency was calculated as follows

$$\begin{array}{c} \text{Encapsulation efficiency}\;(\%)=\frac{\text{mass of the released cargo}}{\text{initial added mass of cargo}}\times 100 \end{array}$$

### Stability test in biorelevant media

Biorelevant media were prepared according to the protocols by Biorelevant. For the preparation of 0.9 liters of FaSSGF, 33.1 g of FaSSGF buffer concentrate was added to 865.7 g of Milli-Q water. Then, 0.054 g of FaSSGF powder was added to the buffer. The FaSSGF was stirred until dissolved and ready to use. The final pH for the FaSSGF was ~1.78. The FaSSIF was prepared by adding 37.48 g of FaSSIF buffer concentrate to 865.0 g of Milli-Q water. Then, 2.016 g of FaSSIF powder was added and stirred until dissolved. The FaSSIF was equilibrated for 2 hours before use. The stability test of plantosomes was conducted by incubating plantosomes in FaSSGF or FaSSIF at 37°C. The incubation of plantosomes in FaSSGF in the presence of pHrodo-dextran (green) and pHrodo-pepsin (red) was conducted under room temperature.

### Release study

FaSSGF and FaSSIF were prepared as described above. Enzyme buffer was prepared by dissolving 2.62 mg of cellulase and 21.46 μl of pectinase in 18 ml of 50 mM citrate buffer (pH = 5). The sequential release study was conducted based on dialysis membrane methods. The plantosomes were prepared by transferring 1.8 ml of CNF stabilized droplets into 6 ml of 200 mM NaCl solution, and then 6 ml of 1 wt % of pectin solution was added. Two milliliters of the plantosomes was sealed in a container with a filter of 5-μm pore size (much larger than the cargo size) and then placed into the buffer with 18 ml of FaSSGF (37°C) and incubated for 1 hour. One hundred microliters of the buffer outside the container was transferred to a 96-well plate every 15 min; an equal amount of the blank buffer was added at the same time. The container was then removed and placed in 18 ml of FaSSIF (37°C) and incubated for 3 hours. One hundred microliters of the buffer outside the container was transferred to a 96-well plate every 30 min. Last, the container was transferred to 18 ml of the enzyme solution (37°C) and incubated for 68 hours. One hundred microliters of the buffer outside the container was transferred to a 96-well plate in an interval of 2 hours for an initial 8 hours, followed by intervals of 12 hours; an equal amount of the blank buffer was added at the same time. The fluorescence intensity of these samples was tested using a plate reader Infinite 200 PRO from TECAN (Switzerland). The excitation wavelength was set at 490 nm, and the emission scan started from 520 to 698 nm.

### Optical microscopy imaging of plantosomes

The structure of plantosomes was observed under an Axio Vert.A1 Light Microscope (Carl Zeiss, Germany). The CLSM images were collected by an LSM 510 UV/Vis (Zeiss, Germany) or an LSM 780 (Zeiss, Germany) with a 40× objective (water immersion objective). The laser excitation wavelengths used were 488 nm (FITC-dextran), 488 nm (Carbotrace 630), 561 nm (Carbotrace 680), 543 nm (Rh-DOPE), 561 nm (Rh-6G), 405 nm (calcofluor-white), 561 nm (pHrodo-pepsin, red), and 514 nm (pHrodo-dextran, green). Images were analyzed with Zeiss Zen 2.6 (blue edition) or Zeiss LSM Image Browser. Carbotrace 630– and Carbotrace 680–stained plantosomes were prepared by incubating plantosomes in the presence of Carbotrace 630 or 680 (0.001 mg/ml) for 2 hours before use. To stain with Rh-6G, dye solution (0.05 mg/ml) was added to a final concentration of 0.008 mg/ml prior to imaging.

### TEM imaging of plantosomes

TEM images were collected on Hitachi, model HT7700 (Hitachi, Japan). Five microliters of the prepared plantosomes was deposited on a TEM grid (200 mesh Formvar/carbon TEM grids) and dried at room temperature. The grid was immersed in 0.1 M NaOH solution for 2 min to remove the lipid layer, followed by washing three times with water. The samples were dried down prior to observation.

### Cytotoxicity tests

Human colon adenocarcinoma cells, Caco-2 cells, were cultured in Dulbecco’s modified Eagle’s medium (DMEM) (GlutaMAX), supplemented with 10% fetal bovine serum, 1% penicillin-streptomycin, and 1% (v/v) non-essential amino acid (NEAA) at 37°C and 5% CO_2_ in culture flasks. Two sets of plates were prepared, one for quantifying cytotoxicity based on the measured LDH activity released from damaged cells and one for live/dead imaging. Approximately 10,000 cells were seeded in replicates of five in 96-well plates and incubated at 37°C and 5% CO_2_ for 2 days. Before treatment, the cells were washed with 150 μl of Gibco FluoroBrite DMEM supplemented with 4 mM l-glutamine and 1% penicillin-streptomycin to remove the residual medium. Supplemented FluoroBrite DMEM was then used throughout the experiments to minimize background absorbance and avoid interference with LDH activity measurements.

Three concentrations of plantosomes were prepared: 20% (*C*_1_), 100% (*C*_2_), and 200% (*C*_3_) of the original protocol, and 1% penicillin-streptomycin was added to prevent the growth of bacteria. Each plantosome concentration was mixed at a 1:2 (v/v) ratio with FluoroBrite DMEM to create the treatment solutions. Likewise, the buffer control was prepared by mixing a blank sample (0.1 M NaCl with 1% penicillin-streptomycin) at 1:2 (v/v) with FluoroBrite DMEM.

Cytotoxicity was measured using the Cytotoxicity Detection Kit^PLUS^ (LDH) from Roche, following the manufacturer’s protocol. Caco-2 cells were incubated with 100 μl of plantosome treatment solutions, buffer control, and FluoroBrite DMEM for live control and lysis control for 6, 24, and 48 hours at 37°C and 5% CO_2_. As background control, 100 μl of supplemented FluoroBrite DMEM was added to the same plate to wells containing no cells and further treated alongside. Fifteen minutes before the end of each treatment time, 5 μl of lysis solution was added to lysis control wells to obtain the high control, representing 100% LDH release. Then, 100 μl of the reaction mixture was added directly to all wells, and after incubation for 15 min at room temperature, 50 μl of stop solution was added. The absorbance was measured immediately at 490 nm using a Thermo Fisher Scientific Varioskan LUX microplate reader. The mean absorbance of the background control was subtracted from all measured absorbance values. The data were then normalized to the high control, and cell viability was calculated as 100% minus normalized LDH release percentage; statistical analysis was carried out with Origin 2024b software. A one-way analysis of variance (ANOVA) with Tukey’s post hoc test was performed to determine the statistical significance versus the live control, and statistically different *P* values were given. No data were excluded from the analysis.

The LIVE/DEAD Cell Imaging Kit was used on the second set of plates according to the manufacturer’s protocol to visualize live (green) and dead cells (red) by confocal fluorescence microscopy with sequential scanning. Cell treatment was performed as described above, and instead of the lysis control, a negative control containing Triton X-100 was added. After removal of the samples, 100 μl of 10% stock solution of live/dead assay in phosphate-buffered saline was added to each well and incubated for 15 min at room temperature in the dark. LIVE/DEAD-stained Caco-2 cells were observed using a Leica TCS SP8 multiphoton microscope equipped with LAS X 3.5.7.23225 software using sequential scanning at 488/500 to 530 nm Ex/Em for live cells and 570/585 to 615 nm Ex/Em for dead cells.
