# Supplementary material for: Plant cell–inspired colon-targeted cargo delivery systems with dual-triggered release mechanisms
Source: Sci Adv. 2025 May 14;11(20):eadt2653. doi: 10.1126/sciadv.adt2653 (PMC12077510; doi:10.1126/sciadv.adt2653)
Supplement: Supplementary file 1 — Figs. S1 to S21 Legend for movie S1 [file sciadv.adt2653_sm.pdf]

Science Advances
AAAS

Supplementary Materials for

Plant cell–inspired colon-targeted cargo delivery systems with dual-triggered release mechanisms

Anran Mao *et al.*

Corresponding author: Anran Mao, anranmao@kth.se; Line Hagner Nielsen, lihan@dtu.dk; Anna J. Svagan, svagan@kth.se

Sci. Adv. **11**, eadt2653 (2025)
DOI: 10.1126/sciadv.adt2653

The PDF file includes:

Figs. S1 to S21
Legend for movie S1

Other Supplementary Material for this manuscript includes the following:

Movie S1

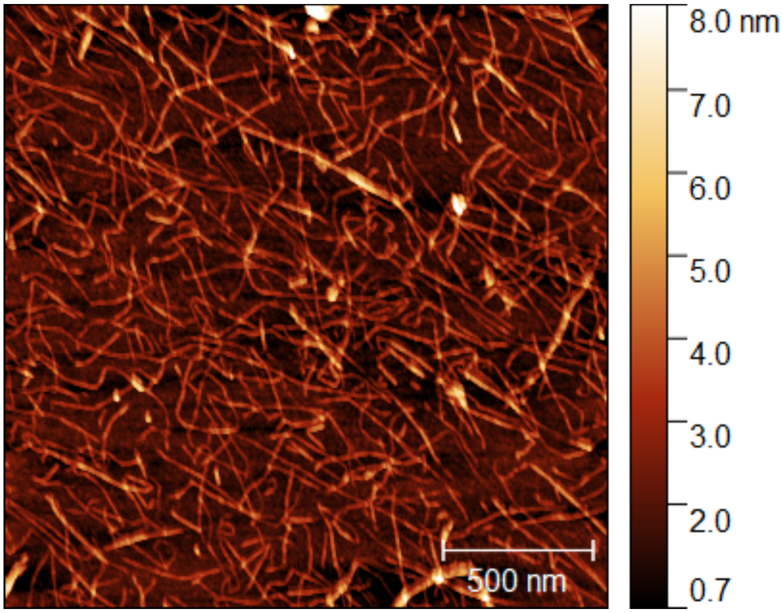

Fig. S1 Atomic force microscopy (AFM) image of the CNFs.

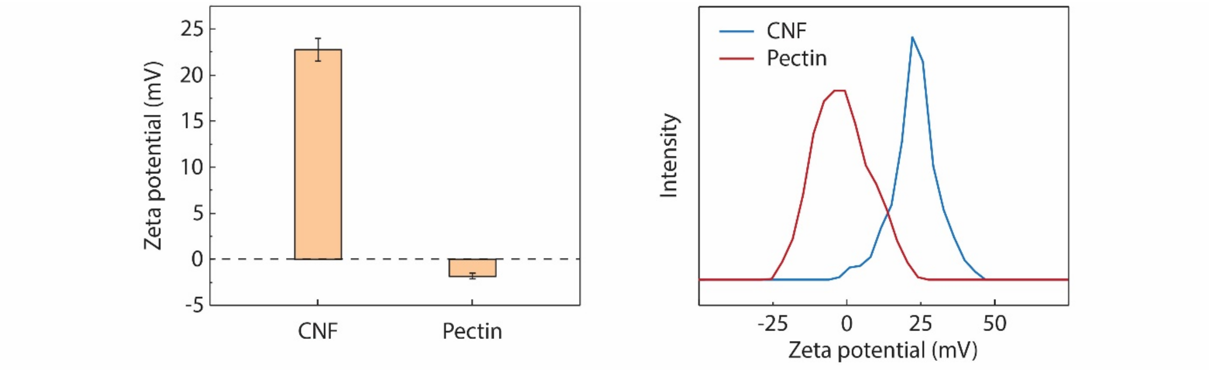

Fig. S2 Zeta potential of CNF and pectin.

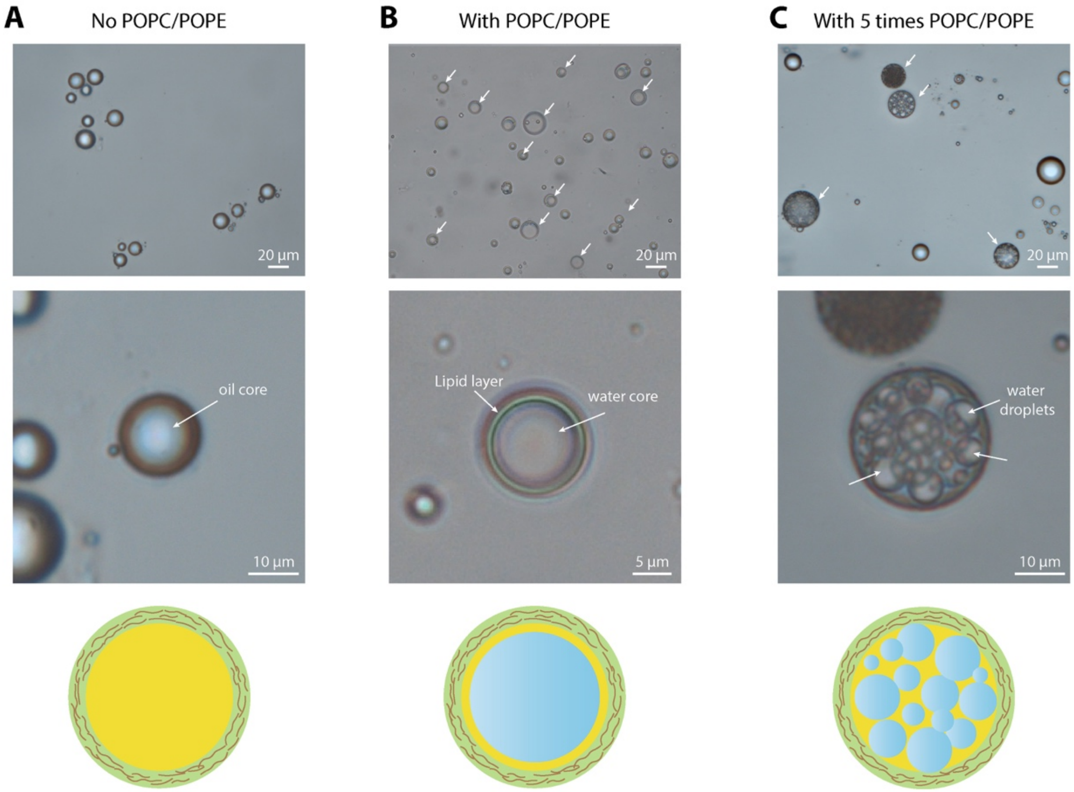

Fig. S3 Structure of capsules with different POPC/POPE content. (A) Microcapsules with oleic acid core when POPC/POPE is not introduced. (B) Plantosomes with a large water core generated after 500 µL of 1 mM/0.2 mM POPC/POPE is introduced. (C) Several multi-compartments with water cores obtained after introducing 500 µL of 5 mM/1 mM POPC/POPE.

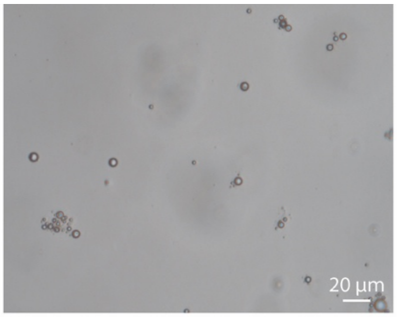

Fig. S4 Capsule sizes (6.0 ± 2.1 µm) obtained with a 0.3 wt% CNF suspension.

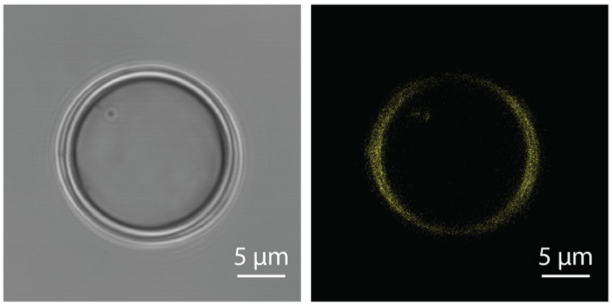

Fig. S5 The bright field image and corresponding CLSM image of plantosome stained with Carbotrace 630.

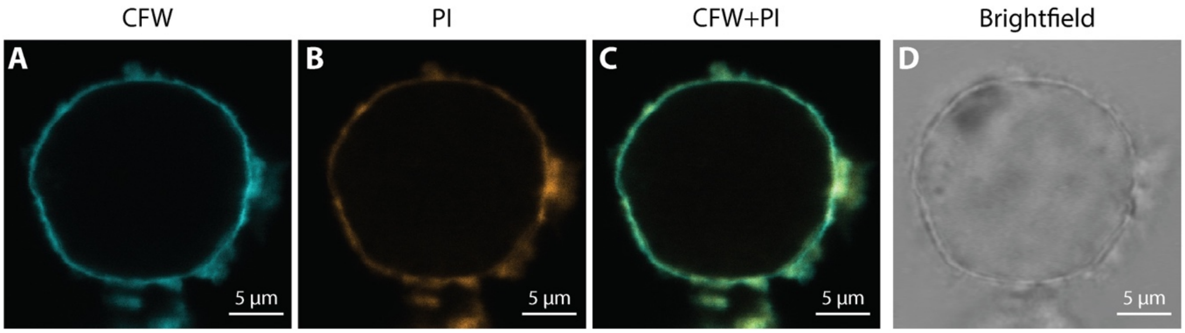

Fig. S6 CLSM images of a plantosome shell stained with Calcofluor-white (CFW, turquoise) and Propidium Iodide (PI, orange). The lipids were removed by increasing to alkaline pH with 0.1 M NaOH prior to staining.

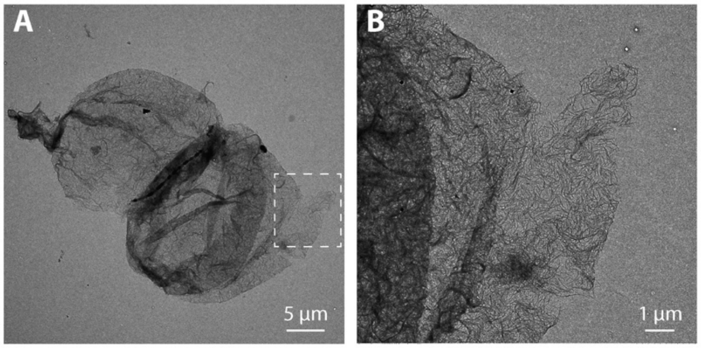

Fig. S7 TEM images of CNF/pectin shells of plantosomes.

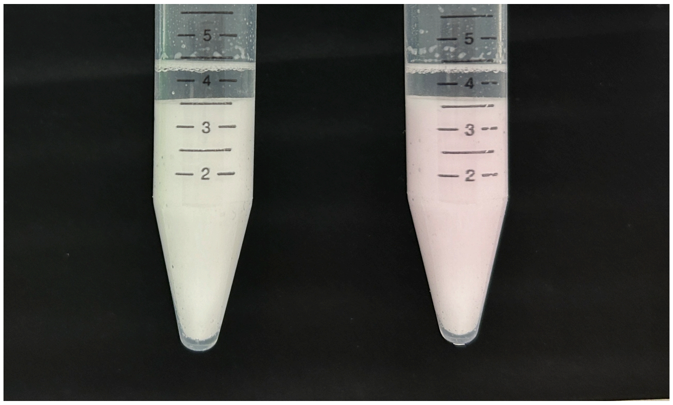

Fig. S8 Photos of emulsion after settling down. (left: blank plantosomes, right: plantosomes with labeled lipids)

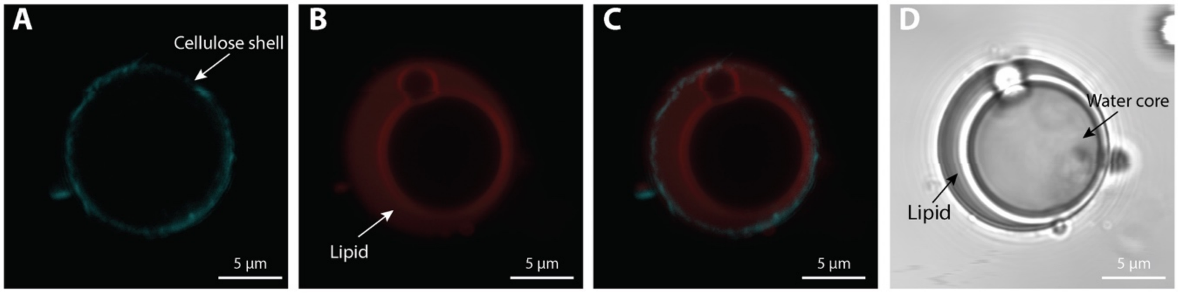

Fig. S9 Colocalization CLSM images of the lipid layer (Rh-DOPE, red) and the cellulose/pectin shell (CFW, turquoise) in a plantosome.

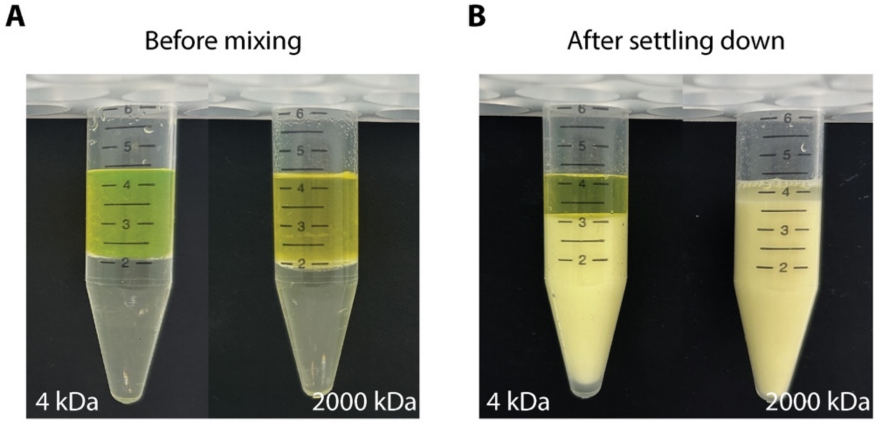

Fig. S10 Photos of cargo-loaded plantosomes during preparation. (A) FITC-dextran loaded plantosomes before mixing. (**B**) FITC-dextran loaded plantosomes after mixing and settling down.

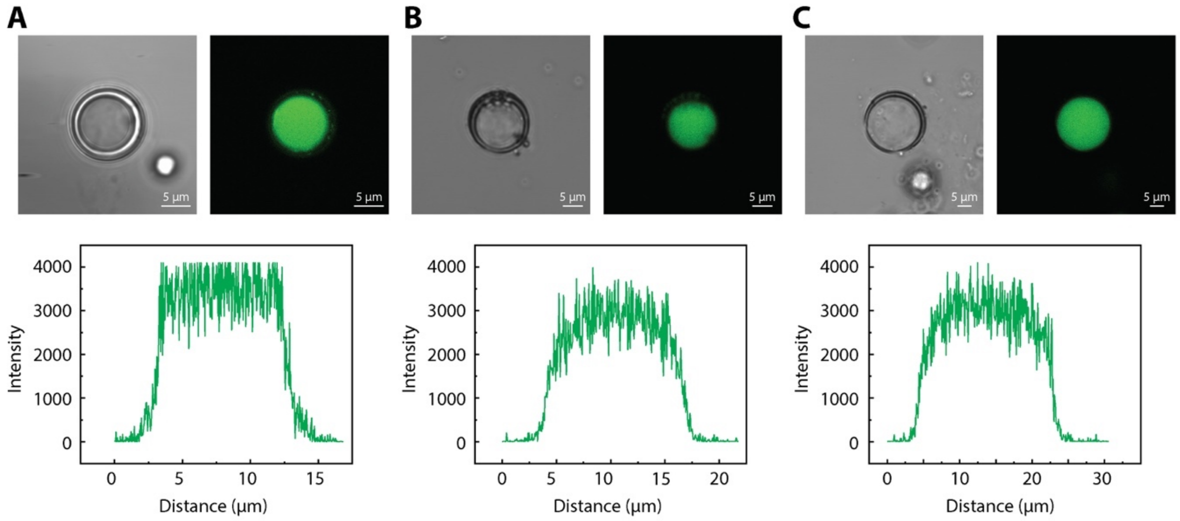

Fig. S11 CLSM images of 4 kDa FITC-dextran loaded plantosomes during dialysis. (**A**) Before dialysis. (**B**) Dialysis for 1 day. (**C**) Dialysis for 7 days.

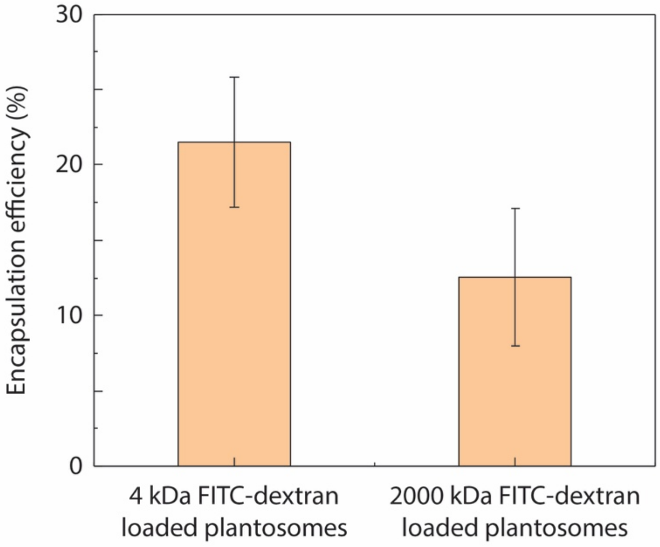

Fig. S12 The encapsulation efficiency of plantosomes.

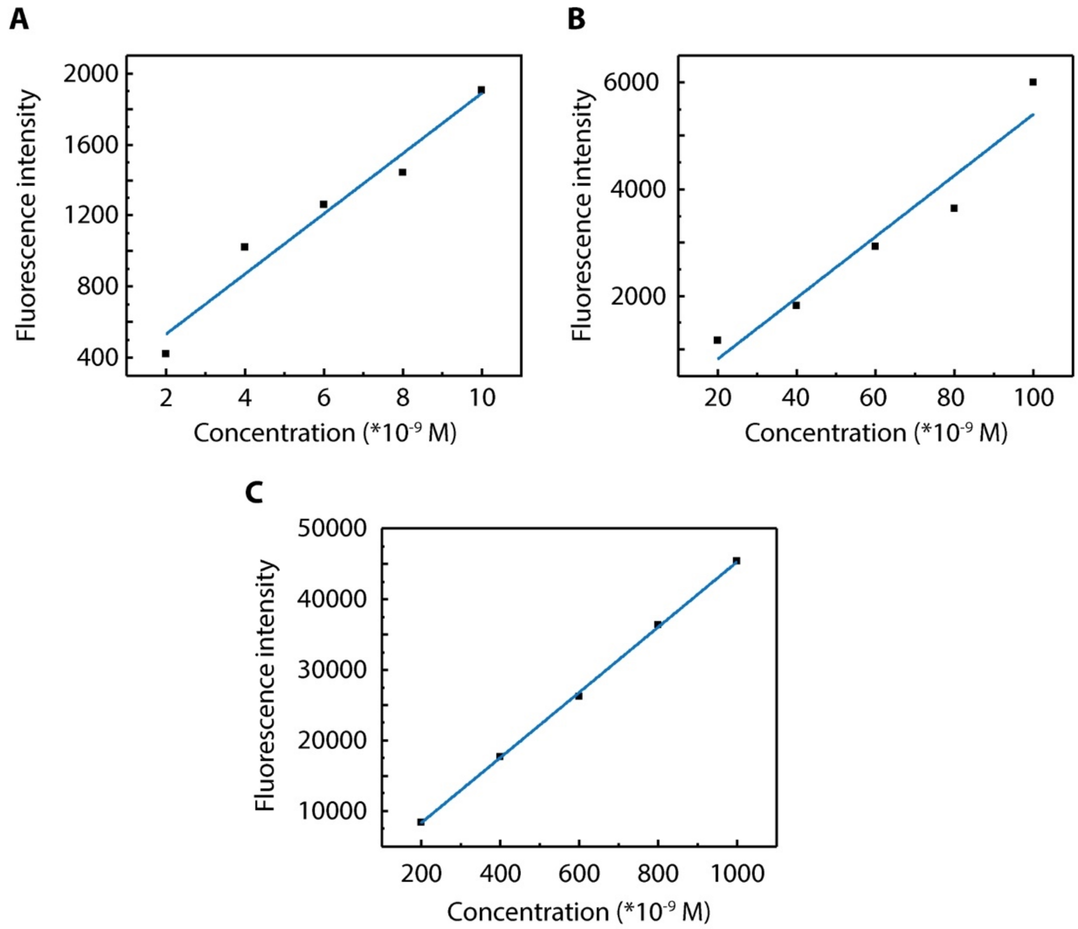

Fig. S13 The standard curves correlating the fluorescence intensity with concentration of 4 kDa FITC-dextran.

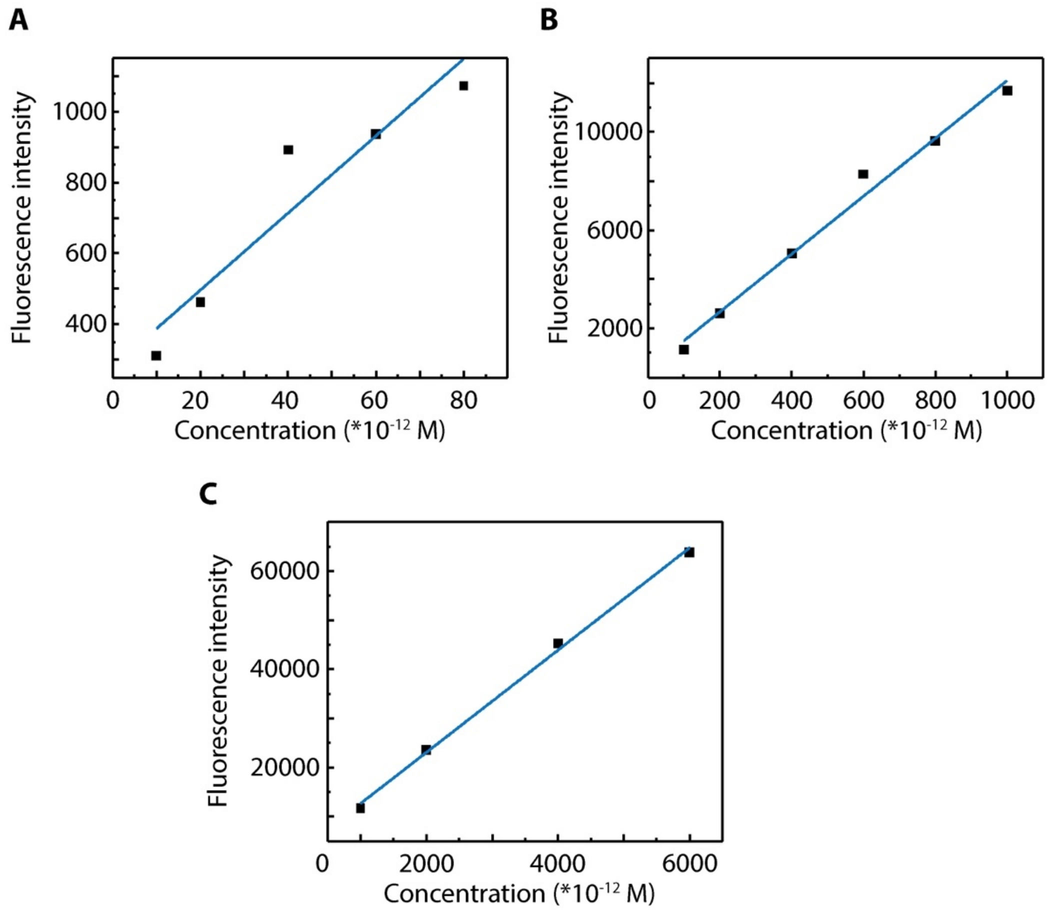

Fig. S14 The standard curves correlating the fluorescence intensity with concentration of 2000 kDa FITC-dextran.

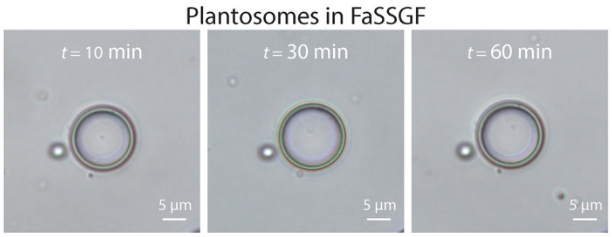

Fig. S15 Stability test of plantosomes in Fasted State Simulated Gastric Fluid (FaSSGF) at 37 ºC.

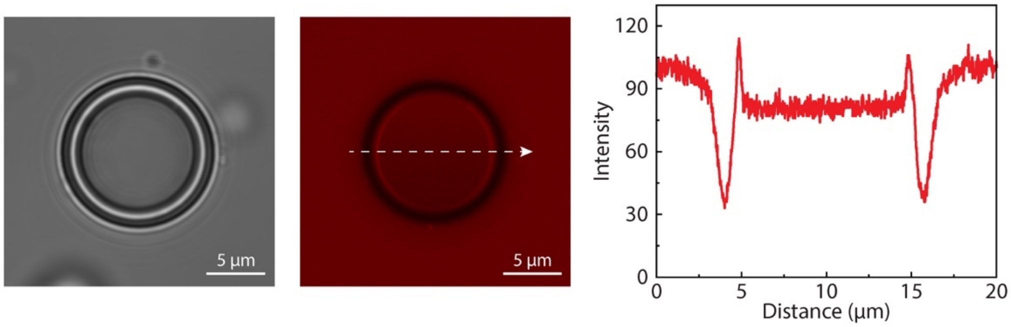

Fig. S16 Bright field and CLSM images of a plantosome incubated in FaSSGF for 30 min in presence of 0.02 mg/mL pHrodo iFL red STP ester (red).

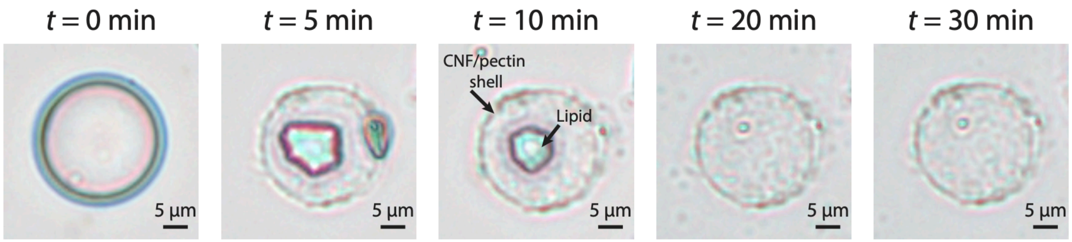

Fig. S17 A plantosome incubated in FaSSIF for 30 min.

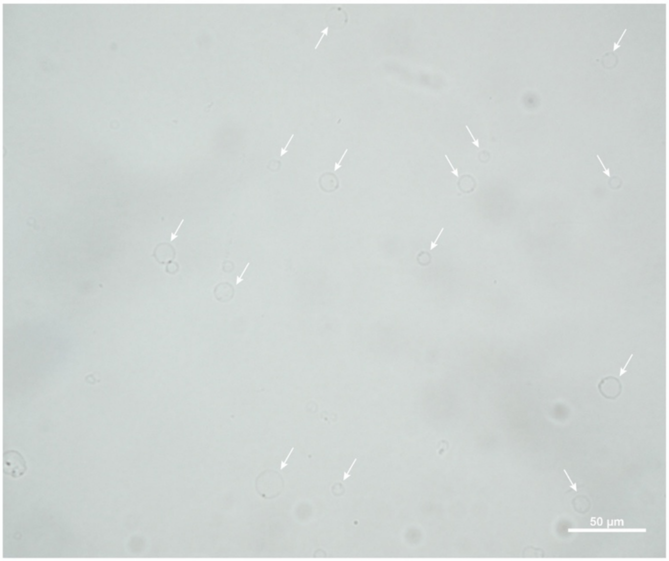

Fig. S18 The bright field image of the CNF/pectin shells after lipids removal.

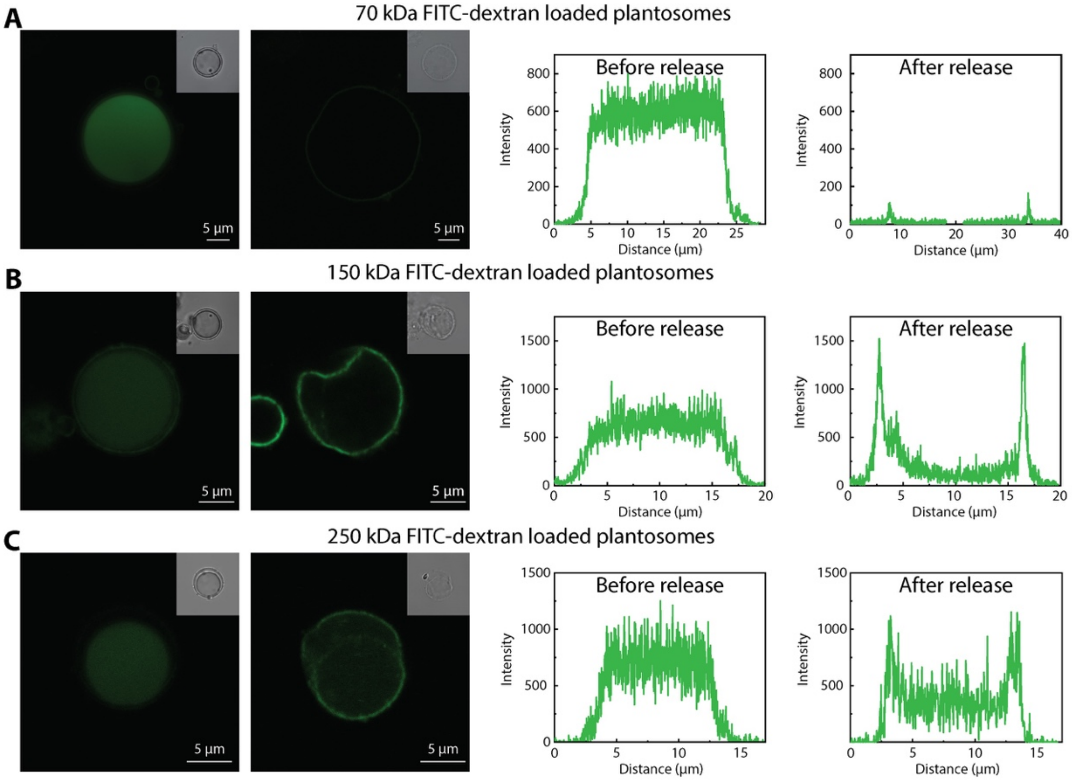

Fig. S19 The release behavior of 70, 150, 250 kDa FITC-dextran-loaded plantosomes in FaSSIF.

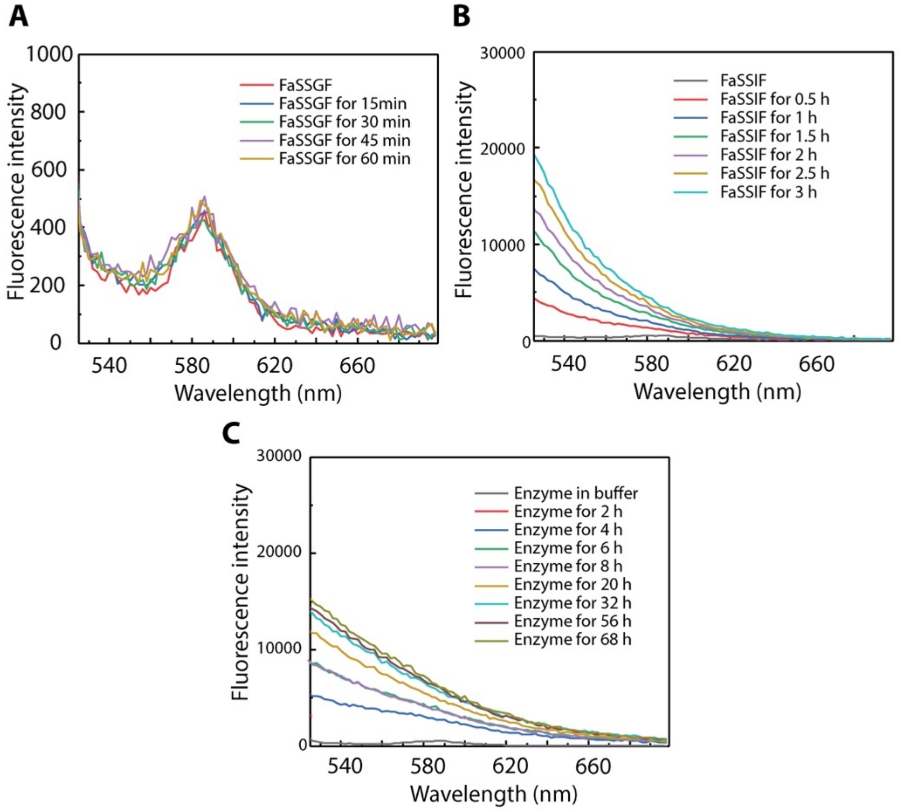

Fig. S20 The emission spectrum of released samples in release study of 4 kDa FITC-dextran loaded plantosomes. (**A**) In FaSSGF. (**B**) In FaSSIF. (**C**) In buffer with enzyme.

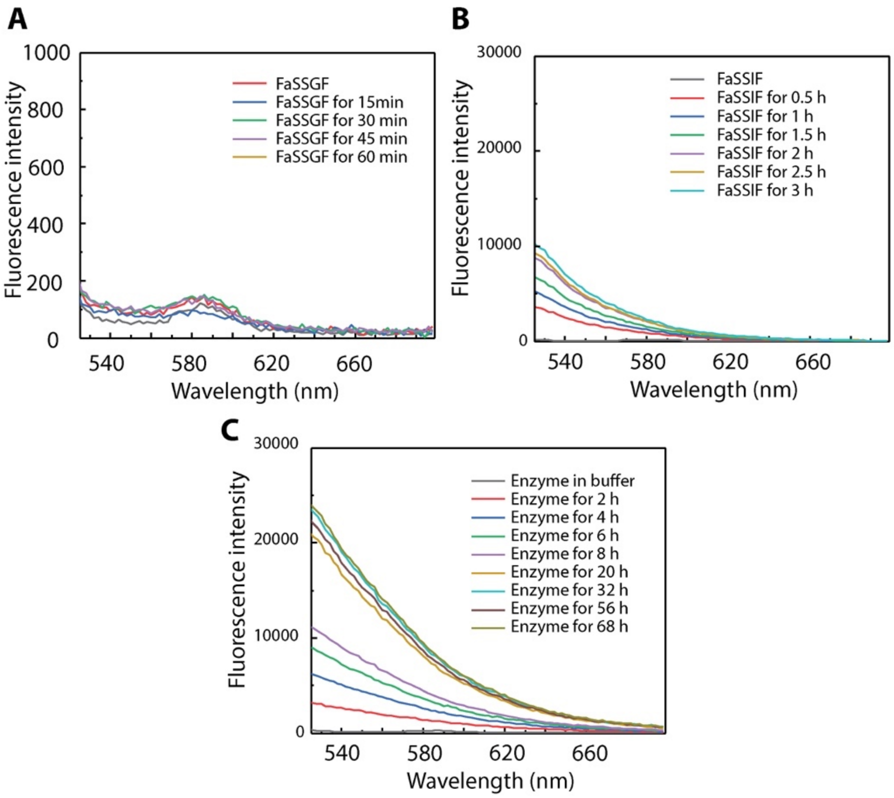

Fig. S21 The emission spectrum of released samples in release study of 2000 kDa FITC-dextran loaded plantosomes. (**A**) In FaSSGF. (**B**) In FaSSIF. (**C**) In buffer with enzyme.

Movie S1.

In-situ observation of plantosomes incubated in FaSSIF.
